# Iodine Nutrition in Emilia-Romagna Region, Italy: A Successful Story of Prevention of Iodine Deficiency Disorders

**DOI:** 10.2174/0118715303427490260109050556

**Published:** 2026-06-10

**Authors:** Domenico Meringolo, Daniela Rotondi, Davide Bianchi, Maria Dolores Scarfoglio, Giovanni Sorrenti, Angela Guaragna, Ramini Remo, Michele Mercuri, Michela Polazzi, Antonella Olivieri, Simona De Angelis

**Affiliations:** 1 Endocrinology Unit, Ramazzini Institute, Bologna, Italy;; 2 Department of Cardiovascular and Endocrine-Metabolic Diseases and Aging, Italian National Institute of Health, Rome, Italy;; 3 Endocrinology Unit, Bentivoglio Hospital, AUSL Bologna, Bentivoglio, Italy

**Keywords:** Iodine prophylaxis, iodine deficiency disorders, iodized salt, Law 55/2005, micronutrient, metabolism

## Abstract

The Emilia-Romagna region, Italy, achieved iodine sufficiency many years before the approval of Law 55/2005, which introduced a nationwide salt iodization program voluntarily in the country. Indeed, more than 10 years before the law, the Emilia-Romagna region implemented a local program of iodine, carrying out information campaigns to promote the use of iodized salt in the population. Epidemiological studies conducted between 1998 and 2005 in this region showed a progressive increase in the use of iodized salt (IS) and a median urinary iodine concentration >100 µg/L in almost all the investigated towns and villages. In 2005, when the national Law 55/2005 was approved, the prevalence of IS consumers in the Emilia-Romagna region was 60%, while it was only 30% in the rest of the country. Further studies conducted between 2011 and 2019 confirmed an adequate iodine intake in the population and the disappearance of goitre in schoolchildren, demonstrating the sustainability of the regional iodine prophylaxis program. Of note, the success obtained in this region is the result of a close collaboration between local politicians, health services, school authorities, families, and salt producers that, in the absence of dedicated funds, moved voluntarily to spread knowledge on the importance of using IS. Multiple and reiterated “on-field” initiatives experienced at the local level yielded awareness in the population on the importance of IS, suggesting that tight cooperation among stakeholders is successful in promoting large-scale programs of prevention.

## 1 INTRODUCTION

The Emilia-Romagna region achieved iodine sufficiency many years before the approval of the national Law 55/2005, which in 2005 introduced a nationwide salt iodization program voluntarily in Italy. The success obtained in this region is the result of a process of close collaboration between local politicians, health services, school authorities, families, and salt producers who have jointly acted to prevent iodine deficiency disorders in the region.

In this narrative review, we describe the most important steps taken to achieve iodine sufficiency in this Italian region. The most representative studies conducted over the years in the Emilia-Romagna region have been selected, and data showing the progressive improvement of iodine nutrition in the population have been synthetized and commented.

## 2 IODINE NUTRITION AND IODINE DEFICIENCY DISORDERS

Iodine is a micronutrient that is essential for the thyroid gland to produce the thyroid hormones (TH), thyroxine, and triiodothyronine [1]. By modulating key cellular processes (*i.e.*, proliferation, differentiation, apoptosis, and metabolism), TH affect multiple body tasks from the early stages of prenatal life (physical, neurological, and intellectual development) to adulthood, during which TH regulate metabolism, thermogenesis, feeding, memory/learning abilities, and cardiovascular and reproductive functions. An adequate iodine intake is therefore essential to maintain lifelong thyroid homeostasis [1, 2].

The daily dietary iodine intake recommended by World Health Organization (WHO), United Nations Children’s Emergency Fund (UNICEF), and International Council for the Control of Iodine Deficiency Disorders (ICCIDD, now Iodine Global Network) to guarantee an appropriate TH biosynthesis is 90 μg for preschool children (0 to 59 months), 120 μg for schoolchildren (6 to 12 years), 150 μg for adolescents (above 12 years) and adults, 250 μg for pregnant and lactating women [3]. Failure to meet these requirements may cause several adverse effects on human health known as iodine deficiency disorders (IDD) [4]. IDD may occur at all ages, from the early stages of foetal life to adulthood (Table **1**) [5]. The detrimental effects of an insufficient maternal intake of iodine on the development and maturation of the foetal brain are of particular relevance [6, 7].

Seafood and saltwater fish are the most relevant iodine sources [8]. In fact, marine fauna and flora accumulate large amounts of soluble iodine from seawaters, while fish from rivers or lakes usually have a low content of iodine [9, 10]. Milk, dairy products, and eggs are other important dietary sources of iodine [11]. It has been calculated that our eating habits meet only 50-60% of the daily iodine requirement in adults [12]. This issue is particularly relevant in certain conditions, such as pregnancy and lactation, when the iodine requirement is nearly double [13, 14]. The requirement of a mother for iodine is increased during pregnancy as a result of an increased requirement for thyroxine to maintain normal metabolism in the mother, a transfer of thyroxine and iodide from the mother to the foetus, and a greater than normal loss of iodide through the kidneys due to an increase in the renal clearance of iodide [15, 16]. During lactation, the physiology of thyroid hormone production and urinary iodine excretion returns to normal, although iodine is concentrated in the mammary gland for excretion in breast milk [17]. However, because of the need to ensure the infant gets enough iodine from breast milk to build reserves in the thyroid gland, an intake of 250 µg of iodine per day is also recommended in lactating women [18].

The epidemiological criteria established by WHO to evaluate the prevalence and severity of iodine deficiency in a specific population refer to the median urinary iodine concentration (UIC) in spot urine samples, along with the presence of goitre in schoolchildren [3]. A geographic area is considered iodine sufficient when the median UIC in schoolchildren is between 100 and 299 μg/L, and goitre prevalence in schoolchildren is below 5% [3, 19]. UIC is a well-accepted, cost-efficient, and easily obtainable indicator for iodine status. Since the majority (>=92%) of dietary iodine is absorbed and in healthy iodine-replete adults >90% is excreted in the urine within 24-48 h [20-22], UIC is considered a sensitive marker of iodine intake which can reflect recent changes in iodine status [23].

Urinary iodine values in schoolchildren are usually expressed as µg iodine/L based on the assumption that the average urinary volume is 1 L/day [11, 24]. In the past, urinary iodine in schoolchildren was also expressed as a function of creatinine (urinary iodine µg/g creatinine). Expressing the urinary iodine concentration as a function of urinary creatinine is useful to minimize the variations caused by variation in urinary volume, as creatinine represents a marker of kidney function [11]. Currently, this correction is used to express urinary iodine concentration in pregnant women and adults showing average urine volume greater than 1 L [11].

A second indicator of iodine status is the goitre rate in schoolchildren. Goitre is an adaptive process caused by an increased stimulation of the thyroid by thyrotropin due to an insufficient supply of dietary iodine. In an effort to maximize the utilization of available iodine, the thyroid gland adapts to an insufficient iodine supply by means of increasing the trapping of iodide [25]. This results in the accumulation within the gland of a larger percentage of the ingested exogenous iodide and a more efficient reuse of iodide directly released by the thyroid or generated by the degradation of TH [25-27]. In endemic areas, although thyroid size decreases in response to an increase in iodine intake, thyroid volume and goitre rate may not return to normal for months or years after correction of iodine deficiency [28]. Therefore, children must be born and raised in an iodine-sufficient environment to reduce the prevalence of goitre to a percentage <5%. For this reason, the prevalence of goitre is considered an indicator of long-term iodine intake [24, 29].

To eradicate IDD, WHO recommends salt iodization as the preferred strategy [3]. Water and bread have occasionally been used, but are clearly not feasible in areas without an extensive piped water supply or among populations that do not universally and daily consume bread. In addition, the fortification of water is expensive, considering the small amount that is used for drinking or the preparation of food. The distribution of iodide tablets has not proved successful owing to difficulties of organization and lack of acceptability. In most parts of the world, the iodination of salt is clearly the best method of goitre control. Salt is consumed daily by everyone and is therefore a most practical vehicle for iodine supplementation [23]. It is worth noting that a salt intake of less than 5 grams (approximately 2 g sodium) per (adult) person per day is recommended by WHO for the prevention of cardiovascular diseases, the leading cause of death globally [30]. For children, the level of salt intake should be adjusted downward based on their energy requirements according to age [31]. WHO has also suggested that the amount of iodine to fortify salt should be adjusted depending on observed sodium intake and surveillance of iodine status in the population [30, 32]. Therefore, policies to eliminate IDD through universal salt iodization and policies to reduce sodium intake to decrease blood pressure and the risk of cardiovascular diseases are considered compatible and can follow a parallel run [32].

## 3 IODINE NUTRITION IN ITALY

Iodine deficiency was first described as a cause of goitre in the early 19^th^ century [33]. Since then, many epidemiological studies have been conducted worldwide reporting iodine status in distinct countries and results of iodine supply at the population level [34, 35]. In Italy, epidemiological studies showing the presence of iodine deficiency both in mountain and coastal areas have also conducted [36, 37].

In our country, the production of salt iodized with 15 mg iodine/kg salt was formally authorised in 1972 [38]. The distribution of iodized salt started selectively in endemic regions, although five years later it was extended to the whole country [39]. However, at that time, the sale of iodized salt was not mandatory and only a small proportion of the Italian population (3%) started using iodized salt [40]. Nevertheless, in the period 1979-1999, local and transient iodine prophylaxis programs were implemented in some Italian regions. Emilia-Romagna region (described below), Sicily and Tuscany regions, as well as the Bolzano autonomous province realized the most effective local programs. Specifically, in 1979 the introduction of iodine into a public water supply was tested in Troina, a town of about 13,000 inhabitants in the North-East part of the Sicily region, where the prevalence of goitre in schoolchildren was 55% and the daily mean urinary iodine excretion (UIE) in non-goitrous adults was 40.7±2.6 μg/L. Four years of iodination of water led to a dramatic reduction of goitre prevalence in children (6%) and the mean daily UIE increased to 85.6±6.5 μg/L [41]. Iodinated water was well tolerated by the population and no adverse effects were observed. However, this method of iodine prophylaxis was abandoned because it could be applied only in areas where a single-source water supply was active [41].

In 1981, a successful voluntary salt iodization program was introduced in the Autonomous Province of Bolzano, the northernmost province of the Trentino region [42]. One year later, a survey conducted on 3,109 schoolchildren showed a prevalence of endemic goitre of 23.6% and a mean UIE of 10.2±8.0 μg/L. Based on these results, an extensive mass-media informative campaign was performed, and after that, more than 50% of the Bolzano population adopted iodized salt. In a follow-up study conducted in 1990, the goitre prevalence decreased to 1.6% and the mean UIE increased to 137.1±104.4 μg/L [42].

In 1981, a smaller-scale but equally successful local iodization program was also introduced in the Tuscan Apennines, Central Italy [43]. Ten years later, a study conducted on 307 schoolchildren residing in two villages (Vagli di Sotto and Castiglione) of this area showed a reduction of the prevalence of goitre in schoolchildren from 60% to 8% and an increase of mean UIE from 47±22 μg/g to 130±73 μg/g of creatinine, underlining the effectiveness of the use of iodized salt in this area [43].

Based on the results of these local iodine prophylaxis programs, in 1991 a new Ministry Decree increased the iodine content of fortified salt from 15 to 30 mg/kg. Furthermore, potassium iodate (more stable) in addition to or in substitution of potassium iodide was introduced [44]. Nevertheless, the sale of iodized salt in food shops and supermarkets was not mandatory yet.

In 2005, a nationwide salt iodization program on a voluntary basis was finally implemented by law (Law n. 55/2005), to prevent endemic goitre and other iodine deficiency disorders [45]. The law requires that iodine be added to coarse and table salt at 30 mg/kg (permitted range 24–42 mg/kg) as potassium iodate and establishes that the availability of iodized salt in food shops and supermarkets is mandatory, whereas non-iodized salt can be sold only on customers’ request. The law also permits the use of iodized salt in communal eating and food industries [45].

In 2009, a nationwide monitoring program of universal salt iodization was implemented, and the Italian National Observatory for Monitoring Iodine Prophylaxis (OSNAMI) was established at the Italian National Institute of Health to evaluate the efficiency and effectiveness of the nationwide program of iodine prophylaxis [46].

After the approval of Law 55/2005, the first nationwide OSNAMI surveillance conducted in 10 Italian regions (period 2007-2012) showed that only 3 regions were iodine sufficient [47]. These regions were Emilia-Romagna, Tuscany, and Sicily, where local iodine prophylaxis programs had been previously implemented. Different results were obtained by the following OSNAMI surveillance, conducted in the period 2015-2019 on 4,233 schoolchildren residing in 10 regions representative of Northern, Central, and Southern Italy. This surveillance finally showed an adequate iodine nutritional status in all the studied regions, included Emilia-Romagna region [48, 49]. Specifically, in the period of observation 74.9% of Italian families used iodized salt. Moreover, iodized salt was also used in 78% of the Italian school canteens. Consistent with these data, the national median value of UIC in schoolchildren was 124 µg/L and the prevalence of goitre 2.2%, with no significant difference between urban and rural areas (1.9% and 2.6% respectively), showing the disappearance of endemic goitre in childhood [48].

## 4 IODINE STATUS IN THE EMILIA-ROMAGNA REGION BEFORE THE IMPLEMENTATION OF THE NATIONWIDE PROGRAM OF IODINE PROPHYLAXIS

Since the 1980s large rural and urban areas have been investigated in the Emilia-Romagna region to verify the success of the local iodine prophylaxis program. Fig. (**1**) shows the geographical distribution of the four provinces representative of the Western (Parma), Central (Reggio-Emilia, Bologna) and South-Eastern (Forlì-Cesena) part of the Emilia-Romagna region where epidemiological investigations have been conducted. The most important results of these studies are reported in Table **2**.

In 1983, a study conducted on 450 schoolchildren in Parma province (Western area of the region) revealed a mean (±standard error) UIC of 72.2±2.5 μg/g creatinine and a goitre prevalence of 54% by palpation [50]. Based on these findings, in 1991 a local program of iodine prophylaxis with iodized salt was introduced in that part of the region. This program was supported by an informative campaign through local newspaper articles, television spots, and local conferences, encouraging the population to consume iodized salt. Iodized salt was available in food stores and, on one occasion, it was distributed free of charge to schoolchildren [51]. In order to verify the effectiveness of this program, a sample of schoolchildren (n=158, age 11-12 years) was monitored and UIC was measured before and after implementing iodine prophylaxis. Six months after the onset of this program, mean UIC rose in children from 83.1±5.1 to 100.6±4.9 μg/g creatinine (*p*<0.01). In addition, it was found that UIC was significantly higher in children who used iodized salt regularly (137.2±9.6 μg/g creatinine) than in those who used it occasionally (85.2±5.2 μg/g creatinine) and who did not use iodized salt at all (75.7±19.5 μg/g creatinine) [51].

In 1986, a large epidemiological study was conducted in the province of Forlì-Cesena (South-Eastern area of the region) on 8,000 schoolchildren. The results showed iodine deficiency with a prevalence of goitre of 43% and a median UIC of 45 µg/L [52].

In the 1990’s new epidemiological studies were conducted in the provinces representative of the Central area of the Emilia-Romagna region. In 1998, 1,020 children aged 12-14 years and residing in Reggio-Emilia province were evaluated. Overall, the mean UIC was 108.7±79.8 µg/L, although the prevalence of goitre assessed by palpation was 26.2% [53].

In 1991, a survey was carried out in Bologna province to verify the prevalence of goitre and the presence of iodine deficiency in relation to different geographical areas identified as flat (Castelguelfo) and mountain (Castel del Rio e Casalfiumanese) of Bologna province. Three hundred and eighty- seven schoolchildren were evaluated. Overall, the median UIC was 65 µg/L, indicative of iodine deficiency. The prevalence of goitre assessed by palpation was 18.5% in mountain areas and 4.6% in flat areas [52].

In 1994, a larger study was conducted to verify the prevalence of goitre and the presence of iodine deficiency in 740 schoolchildren aged 6-15 years residing in flat, hilly, and mountain villages (Baricella, Castello di Serravalle, San Benedetto Val di Sambro, respectively) of Bologna province. The study involved 740 schoolchildren aged 6-15 years. An overall prevalence of goitre on thyroid ultrasound of 4.75% (4.2%, 4.81%, and 5.11% in flat, hilly and mountainous villages, respectively) and a median UIC of 87.3 µg/L, indicative of a mild iodine deficiency, were found [52]. An educational project based on an informative campaign to promote the use of iodized salt on a regular basis, and specifically directed to families of schoolchildren, was then launched in Bologna province. The program was developed in cooperation with the local institutions and school authorities and was supported by salt producers. Moreover, the promotion was reiterated throughout local newspapers, TV and radio broadcasts.

Some years later in 1998, a further epidemiological study was conducted in Bologna province on 233 schoolchildren residing in mountain municipalities of the Apennines (Castel d’Aiano, Gaggio Montano, Pietracolora) and 175 children recruited as a control group and residing in the urban area of ​​Bologna. The prevalence of goitre in schoolchildren residing in mountainous areas was 18% by palpation with a median UIC of 119 µg/L. In the control group, the incidence of goitre was 6.3% with a median UIC of 159 µg/L [54].

In 2004, an epidemiological survey was repeated on 133 schoolchildren residing in three municipalities (Baricella, Castello di Serravalle, and San Benedetto Val di Sambro) of Bologna province. Overall, a median UIC of 110 µg/L indicative of iodine sufficiency was observed. In addition, a goitre prevalence of 3.46% on ultrasound was found, showing the disappearance of endemic goitre in the Bologna area [52]. In 2005, another epidemiological survey was conducted in the Bologna Apennines area (Castiglione dei Pepoli village) where local interventions aimed at promoting iodine prophylaxis had been performed for long time [52]. Three hundred nineteen schoolchildren were recruited for the study. The results showed a median UIC of 98.5 µg/L and a prevalence of goitre of 4.4% on ultrasound, suggesting a sufficient and long-lasting adequate iodine intake in the population. The analysis of data also showed that 60% of the schoolchildren’s families participating in the study used iodized salt, a percentage which was double that observed in other parts of Italy at that time [47]. These important results obtained in Bologna province can be attributed to a great awareness of the population on the importance of using iodized salt, achieved by means of TV and other information channels [52].

In summary, the epidemiological studies carried out between 1983 and 2005 in the Emilia-Romagna region showed a progressive increase in the use of iodized salt followed by a progressive decrease in goitre prevalence and an increase in UIC median values in the population. These results also showed that Parma, Reggio-Emilia, and Bologna provinces were already iodine sufficient before the approval of the national Law 55/2005, which introduced the nationwide program of iodine prophylaxis. It is worth noting that in the years before the introduction of the nationwide program of iodine prophylaxis (period 1992-2004), most of the Italian regions were still iodine-deficient (Table **3**) [47, 55-60].

## 5 IODINE STATUS IN THE EMILIA-ROMAGNA REGION AFTER THE IMPLEMENTATION OF THE NATIONWIDE PROGRAM OF IODINE PROPHYLAXIS.

In 2011, 6 years after the introduction of the nationwide iodine prophylaxis program, an additional survey was conducted on 300 schoolchildren residing in Bologna province (San Benedetto Val di Sambro village) [61]. A median 24 h UIE of 121 µg/L was found, confirming iodine sufficiency in the Bologna area. Accordingly, a high percentage of household iodized salt use (81%) was also observed at that time [61].

The most recent survey in the Emilia-Romagna region was conducted in the period 2017-2019. This was part of the second nationwide survey on the iodine nutritional status in Italy conducted on schoolchildren by the OSNAMI with the collaboration of the Regional Observatories for Goitre Prevention [48].

The Emilia-Romagna Regional Observatory collected data of 391 schoolchildren (197 boys and 194 girls) aged 11-13 years. The study aimed to verify the sustainability of an adequate iodine supply in the rural areas of Bologna province (n=130 schoolchildren residing in Bentivoglio village) as a control area, and in Forlì-Cesena province (n=261schoolchildren residing in San Mauro Pascoli village) where no further surveys had been performed since 1987 [52]. The results of this survey demonstrated the efficiency of the iodine prophylaxis program in both provinces. Specifically, 67.6% of schoolchildren’s families reported routine use of iodized salt, with no significant difference between Bologna and Forlì-Cesena provinces (Table **4**).

Moreover, the analyses of data on UIC confirmed the iodine sufficiency in this part of the region with a median UIC of 130.5 μg/L, and no significant differences between Bologna and Forlì-Cesena areas (113.8 µg/L and 140 µg/L, respectively). The analysis of the prevalence of goitre in schoolchildren showed an overall prevalence of 1.8%, far below the threshold value of 5% indicative of endemic goitre, with no significant differences between Bologna and Forlì-Cesena (1.5% and 1.9%, respectively). These results confirmed the adequate iodine nutrition in the region [61].

## CONCLUSION

The epidemiological studies carried out between 1983 and 2005 in the Emilia-Romagna region showed a progressive increase in the use of iodized salt followed by an increase in UIC median values and a progressive reduction of goitre prevalence in schoolchildren. These results also showed that the Emilia-Romagna region was already iodine sufficient before the approval of Law 55/2005, which introduced the nationwide program of iodine prophylaxis. Further studies conducted by OSNAMI in 2011 and in the period 2014-2019 confirmed iodine sufficiency in the region.

This success is the result of a close collaboration between local politicians, health services (Local Health Units leadership, family physicians and paediatricians, endocrinologists), school authorities (school principals, teaching staff), families, salt producers, managers of canteens, and meal production centres. Over the years, all have moved voluntarily and, even in the absence of dedicated funds, all have done their best to spread knowledge on the importance of using iodized salt, providing a model of cooperation to prevent IDD. Currently, the 2021-2025 Emilia-Romagna Regional Prevention Plan includes a Strategic Objective aimed at improving control activities on the presence of iodized salt in supermarkets and collective catering, demonstrating the continuous commitment of the institutions in ensuring the sustainability of the iodine prophylaxis program in the Emilia- Romagna region [62].

However, even in such a positive context, it is important to underline that changes in diet and lifestyle, as well as the progressive decrease in salt consumption, may jeopardise the maintenance of iodine sufficiency in the Emilia-Romagna region as in the rest of Italy. It is therefore important to provide iterations of informative campaigns on the use of iodized salt and to promote the consumption of iodine-enriched foods. Among these, milk and dairy products provide the largest contribution because the current EU regulations permit a maximum of 5 mg iodine/kg dry matter for dairy ruminants, with 2 mg/kg recommended where possible [63, 64].

Biofortification of vegetables with iodine also offers an excellent opportunity to increase iodine intake. In Italy, several iodized vegetables have been developed by the food industry and are currently available in the Italian market contributing to improve the iodine nutritional status of the population without risks of iodine excess [65]. Of note, the Emilia Romagna region represents an excellence in the production of iodine biofortified potatoes grown in open fields [66]. Another issue is that the use of iodized salt in the food industry is not mandatory in our country. Lastly, the cost of iodized salt in Italy is higher (about +20%) than that of non-iodized salt because of higher production costs [37]. This implies that further efforts should be made to convince the salt producers to lower their costs. At the same time, every effort will be made to guarantee the sustainability of the iodine prophylaxis program and to maintain an efficient surveillance system that constantly monitors the iodine status of the population.

In conclusion, although the small sample size of some of the reported studies may represent a limitation, the large number of analysed studies is a strength of this review article, which provides consistent data on the improvement over time of iodine nutrition in the population and documents the sustainability of the iodine prophylaxis program in the region.

## Figures and Tables

**Fig. (1):**
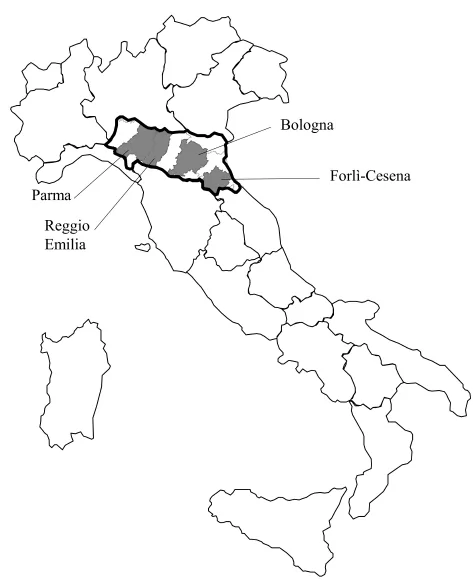
Map of the Emilia-Romagna region showing the provinces with available epidemiological data on iodine supply in humans (grey areas).

**Table 1:** Iodine deficiency disorders, by age group.

**Age Group**	**Health Consequences**
Foetus	Abortion Stillbirth Congenital anomalies Perinatal mortality
Neonate	Infant mortality Endemic cretinism
Child and adolescent	Impaired mental function Delayed physical development
Adults	Impaired mental function Reduced work productivity Toxic nodular goitre, iodine-induced hyperthyroidism Increased occurrence of hypothyroidism in moderate-to-severe iodine deficiency, decreased occurrence of hypothyroidism in mild-to-moderate iodine deficiency
All ages	Goitre Increased susceptibility of the thyroid gland to nuclear radiation

**Note: **Adapted by [5].

**Table 2:** Main results obtained in epidemiological investigations conducted in the Emilia-Romagna region (period 1983-2017).

**Province**	**Period of Observation**	**Number of School Children**	**UIC (µg/L)** **(Mean±SD or Median Value)**	**Goitre Prevalence (%)**
Parma	1983 [50]	450	72.2±2.5*	54 (palpation)
	1991 [51]	158	83.1±5.1* (before iodine prophylaxis)	-
	1991 [51]	158	100.6±4.9* (6 months after iodine prophylaxis)	-
Forlì-Cesena	1986 [52]	8000	45	43 (palpation)
	2017 [52]	261	140	1.5 (ultrasound)
Reggio-Emilia	1998 [53]	1020	108.7±79.8	26.2 (palpation)
Bologna	1991 [52]	387	65	Mountain area 18.5 Flat area 4.6 (palpation)
	1994 [52]	740	87.3	4.7 (ultrasound)
	1998 [54]	233	Mountain area 119 Urban area 159	Mountain area 18.8 Urban area 4.6 (palpation)
	2004 [52]	133	110	3.4 (ultrasound)
	2005 [52]	319	98.5	4.4 (ultrasound)
	2011 [61]	300	121	-
	2017 [52]	130	113.8	1.9 (ultrasound)

Note: *mean±SE; µg/g creatinine. **Abbreviations:** UIC, urinary iodine concentration; SD, standard deviation; SE, standard error.

**Table 3:** Prevalence of goitre and urinary iodine concentration (UIC) in schoolchildren obtained from epidemiological surveys carried out in some Italian regions from 1992-2004.

**Italian Regions**	**Years 1992-2004**	
	**UIC Median (µg/L)**	**Goitre (%)**
Marche [47]	95±25^a^	10
Umbria [55]	94	8
Campania [56]	79	-
Abruzzo [47]	65*	25
Molise [57]	-	30
Basilicata [58]	55	16
Puglia [59]	95	6
Calabria [60]	90±60^a^	5.3-26

**Note:** ^a^mean±standard deviation; *μg/g creatinine.

**Table 4:** Results on knowledge of iodized salt in the survey conducted in Emilia-Romagna in 2017-2019.

-	**Provinces**		-
-	**Bologna** **N=130**	**Forlì-Cesena** **N=261**	**Total** **N=391**
Do you know iodized salt? N. (%)	111 (85)	233 (89)	344 (87)
Do you use iodized salt? N. (yes, %)	88 (68)	175 (67.2)	264 (67.6)
Use of iodized salt >10 years N. (%)	97 (75)	183 (70)	287 (73.3)
Presence in the store N. (%)	118 (91)	220 (84)	342 (87.5)
Do you know what iodized salt is used for? N. (%)			
Prevents goitre	111 (85)	211 (80)	322 (82.5)
Improves the taste of food	7 (5)	14 (5)	21 (5)
Preserves food	4 (3)	6 (2)	10 (2.5)
Not known	8 (6)	30 (12)	38 (10)

## LIST OF ABBREVIATIONS

IS: Iodized Salt
TH: Thyroid Hormones
WHO: World Health Organization
UNICEF: United Nations Children’s Emergency Fund
ICCIDD: International Council for the Control of Iodine Deficiency Disorders
IDD: Iodine Deficiency Disorders
UIC: Urinary Iodine Concentration
UIE: Urinary Iodine Excretion
OSNAMI: Italian National Observatory for Monitoring Iodine Prophylaxis
